# Editorial: Peritoneal metastasis of gastric cancer

**DOI:** 10.3389/fonc.2023.1273551

**Published:** 2023-08-28

**Authors:** Shuyi Wang, Jialin Song, Bin Xiong

**Affiliations:** ^1^ Department of Gastrointestinal Surgery, Zhongnan Hospital of Wuhan University, Wuhan, China; ^2^ Biology Laboratory, Hubei Key Laboratory of Tumour Biological Behaviours, Wuhan, China

**Keywords:** gastric cancer, peritoneal metastasis, tumor microenvironment, CXCL8, FMO family

Peritoneal metastasis is the main cause of death in gastric cancer patients. Despite radical gastric cancer surgery, peritoneal metastasis occurs in about 50% of gastric cancer patients and the overall survival is less than 6 months (1, 2). From the perspective of biological function, gastric cancer peritoneal metastasis (GCPM) is a multistep, multilayered and complex process that is closely related to the microenvironment of the gastric cancer primary site, the peritoneal pre-metastatic microenvironment, and the intraperitoneal microenvironment. Due to the complexity of the peritoneal ecosystem, the mechanism of GCPM remains to be deciphered. Early diagnosis of GCPM relies on imaging techniques, intra-abdominal cytology, and diagnostic laparoscopy. However, due to the presence of undeformed peritoneal cancer nodules and microscopic metastatic foci, peritoneal metastasis cannot be detected in a timely and accurate manner by the conventional diagnostic modalities. The treatment of GCPM is recommended to focus on systemic therapy. Cytoreductive surgery and hyperthermic intraperitoneal chemotherapy (CRS-HIPEC) can be performed according to the patient’s condition, however the recurrence rate is still high (3). Therefore, it is still a major task to explore new mechanisms, new diagnostic methods and new therapeutic means of GCPM, which is deeply explored in this Research Topic To provide new insights for the study of GCPM.

Tumor microenvironment (TME) plays an important role in the pathogenesis of GCPM, Fu et al. reviewed the mechanisms of paracrine or autocrine CXCL8 in TME regulates the development of tumor peritoneal metastasis in multiple pathways. Upregulation of CXCL8 in tumor cells biologically promotes tumor cells EMT, anoikis resistance, angiogenesis, and peritoneal metastasis in the tumor primary microenvironment. In peritoneal microenvironment, CXCL8 promotes peritoneal-associated cells adhesion, and angiogenesis and tumor cells colonization and proliferation. The review concludes by proposing new treatments for developing a series of drugs targeting CXCL8 or CXCR1/2 to slow the progression of peritoneal metastases.

Radiographic techniques are the mainstay of evaluating peritoneal metastases in patients with gastric cancer. Li et al. discussed the important role of Computed tomography (CT) characteristics in the prognosis of GCPM patients through a retrospective study of 66 patients diagnosed as gastric cancer with peritoneal metastasis. This study assessed triple tract dilatation (TTD), extensive lymph node metastasis (ELM), the grade of ascites and overall survival (OS) in patients with GCPM by constructing The CT characteristic-based PM score system, which in turn identified patients who could not benefit from palliative chemotherapy. This study provides a basis for the multi-omics prediction of peritoneal metastasis in patients with gastric cancer.

Tumor markers still have value in diagnosing peritoneal metastases. With the deepening of research, peritoneal-related genes have a certain reference value in predicting peritoneal metastasis in gastric cancer patients. Gong et al. analyzed the role of FMO family in GCPM using bioinformatics. The study identified a 12-gene panel for predicting the peritoneal metastasis (PM) risk signature based on the FMO family and found that FMO is a potential target for diagnosis and treatment of GCPM.

Palliative therapy is the most common treatment modality for gastric cancer patients with peritoneal metastasis. Luo et al. reviewed various approaches to palliative therapy in patients with different advanced stages of gastric cancer. This review compares different palliative therapy methods, combines clinical guidelines and current researches, and presents the selection conditions and considerations of each method, also proposes the combined use of different palliative therapies to improve the therapeutic effectiveness, which help us to select the treatment modality when facing different patients with advanced gastric cancer.

In conclusion, this Research Topic collects reviews and original researches related to peritoneal metastasis of gastric cancer, which aims to describe the current research directions and daunting challenges in terms of mechanism, diagnosis and treatment of GCPM.

## Author contributions

BX: Writing – original draft, Writing – review & editing. SW: Writing – original draft, Writing – review & editing. JS: Writing – original draft.
